# Congenital Hypertrophic Stenosis of the Pylorus

**Published:** 1905-03-25

**Authors:** 


### CONGENITAL HYPERTROPHIC STENOSIS OF THE PYLORUS.

This disease is commonly spoken of as a congenital malady, though in a majority of cases the symptoms do not appear for several weeks after birth. Dr. Still[1] says that the vomiting which marks the lesion, although postponed for a week or two after birth, is almost never later in its appearance than the eighth week of life. The cardinal symptoms are vomiting, wasting, and constipation, with visible peristalsis of the stomach and the presence of a palpable tumour (representing the enlarged pylorus) to the right of the middle line and about midway between the costal margin and the horizontal level of the navel. Dr. Still observes that both the peristalsis and the tumour must be sought on several occasions and with patience, for since the appearance of either depends chiefly on muscular contraction of the stomach, it is not to be supposed that they can be identified at any moment. He considers that the best time to make the examination is either during or immediately after the feeding-time.

As regards treatment, Dr. Still says that a considerable proportion yield to a careful dietary, the aim being to choose a food of little residue, such as whey with egg-albumin, broths, meat-juice, etc. In other cases success may be achieved by daily lavage of the stomach with a solution of bicarbonate of soda and water (2 grains to the ounce). The test of improvement is a gain in weight, and if this cannot otherwise be accomplished there remains operative interference. In the series of cases under notice, nine out of 20 required operation. The operation was in each case a forcible dilation of the pylorus, and of the nine cases seven ultimately recovered.

[1] Lancet, March 11, 1905.

